# Differential silencing of STAT3 isoforms leads to changes in STAT3 activation

**DOI:** 10.18632/oncotarget.28412

**Published:** 2023-04-24

**Authors:** Inbal Shamir, Ilan Tsarfaty, Gidi Paret, Yael Nevo-Caspi

**Affiliations:** ^1^Department of Pediatric Critical Care Medicine, Safra Children’s Hospital, Sheba Medical Center, Ramat-Gan, Israel; ^2^Department of Clinical Microbiology and Immunology, Sackler School of Medicine, Tel Aviv University, Tel Aviv, Israel; ^*^These authors contributed equally to this work

**Keywords:** STAT3, STAT3 isoforms, breast cancer, cancer diagnosis, cancer therapy

## Abstract

Signal transducer and activator of transcription 3 (STAT3) is a transcription factor involved in multiple fundamental biological processes and a key player in cancer development and progression. STAT3 is activated upon tyrosine phosphorylation and is constitutively active in various malignancies; therefore, the expression of pSTAT3 has been recognized as a predictor of poor survival. STAT3 encodes two alternatively-spliced STAT3 isoforms: the full-length STAT3α isoform and the truncated STAT3β isoform. These isoforms have been suggested as the reason for the occasionally observed opposing roles of STAT3 in cancer: an oncogene, on one hand, and a tumor suppressor on the other. To investigate their roles in aggressive breast cancer, we separately silenced each isoform and found that they affect each other’s activation, impacting cell viability, cytokine expression, and migration. Silencing specific isoforms can lead to a more favorable balance of activated STAT3 proteins in the cell. Distinguishing between the two isoforms and their active forms is crucial for STAT3-related cancer diagnosis and therapy.

## INTRODUCTION

Signal transducer and activator of transcription 3 (STAT3) is a transcription factor involved in multiple fundamental biological processes such as proliferation, cell survival, differentiation, migration, metabolism, and immune regulation. STAT3 is activated by tyrosine (Y705) phosphorylation, typically in response to extra-cellular ligands such as cytokines and growth factors. Following activation, STAT3 undergoes dimerization and translocation to the nucleus where it regulates transcription of multiple target genes. STAT3 is also phosphorylated on a serine (S727) residue. This phosphorylation contributes to its maximal transcriptional activity and to its localization and function in the mitochondria and endoplasmic reticulum (ER) [1, 2].

**Figure 1:**
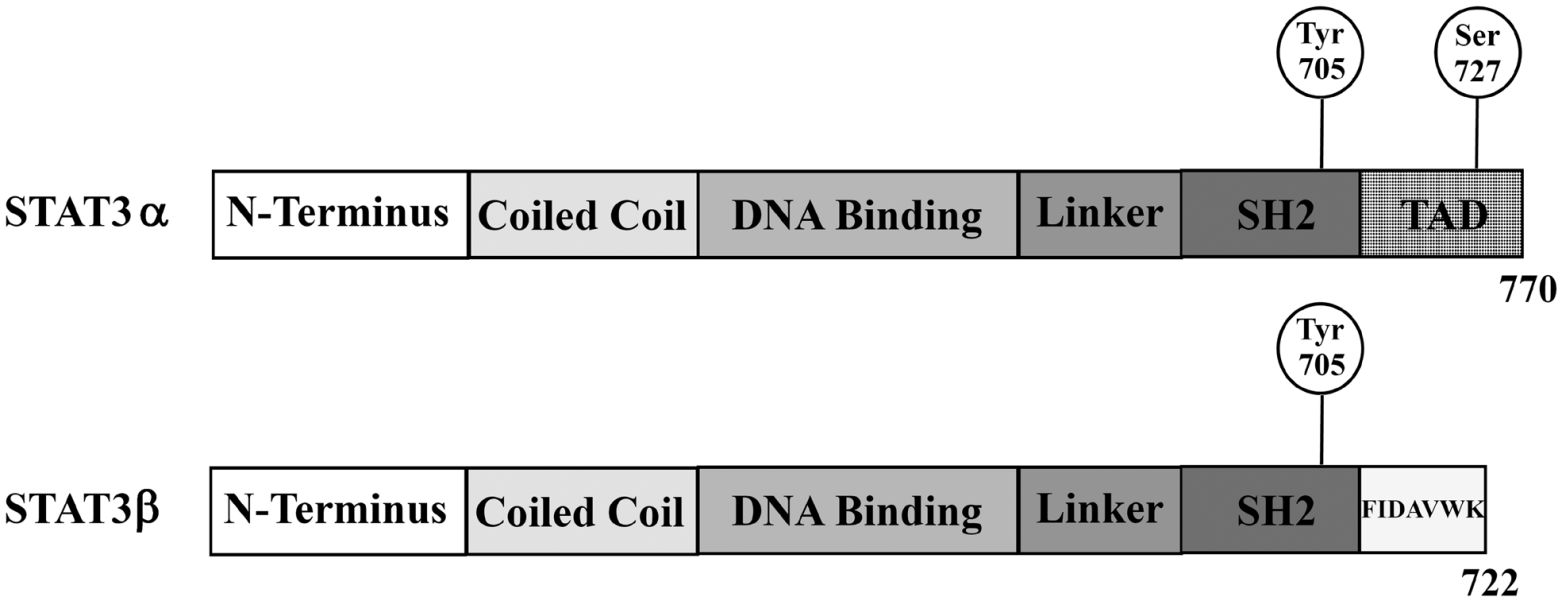
Schematic overview of the two alternatively spliced STAT3 isoforms STAT3α and STAT3β. STAT3β lacks the transactivation domain (TAD) and instead carries seven unique amino acids; Src homology 2 (SH2).

STAT3 is expressed in all cell types. Aberrant regulation of STAT3 has been reported in nearly 70% of cancers [3–5]. It is well-known as a major factor in modulating pro-tumorigenic mechanisms by driving inflammation, cell survival, evasion of apoptosis, invasion and metastasis [2, 3, 6, 7]. In various types of malignancies, it has been shown to be constitutively active and therefore, the expression of pSTAT3 has been recognized as a predictor of poor survival [2]. Indeed, STAT3 is considered an oncogene and as such, the enormous therapeutic potential of STAT3 inhibitors is well recognized. Such inhibitors exert their effect either directly, by regulating the expression of STAT3 or by preventing the formation of functional STAT3 dimers through disrupting phosphorylation of STAT3, or by preventing STAT3 binding to DNA, or indirectly by blocking upstream signaling pathways [8, 9]. However, accumulating evidence from both experimental and clinical studies, suggests that STAT3 may also function as a tumor suppressor. The opposing roles of STAT3 in cancer may be explained by the existence of two alternatively-spliced STAT3 isoforms: the full-length STAT3α isoform (92 kDa) and the truncated STAT3β isoform (83 kDa) (Figure 1). These transcripts encode almost an identical amino acid sequence however STAT3β lacks the C-terminal transactivation domain (55 amino acids) including the serine (S727) phosphorylation site, which is replaced by a tail of seven unique amino acids [2, 3]. Due to the lack of the transactivation domain, many studies refer to STAT3β as a dominant negative factor of STAT3α, although lately, it has become evident that STAT3β has its own set of target genes that is distinct from that of STAT3α [10]. Nevertheless, STAT3α is considered as an oncogene and STAT3β as a tumor suppressor. Recent studies have demonstrated that in order to execute the suppressive role of STAT3, both STAT3α and STAT3β are needed [6, 11–13]. The opposing roles of STAT3 may also be attributed to the different STAT3 dimers: since both isoforms can be activated by tyrosine phosphorylation (Y705), the formation of homo- and heterodimers, which represent the functional STAT3 dimer, can readily occur, however their biochemical and biological properties have been shown to differ significantly [2, 3, 5]. Although both isoforms are co-expressed in all cell types, STAT3α (often referred to as STAT3) is generally expressed at higher levels than STAT3β. Interestingly, the relative amount of these two isoforms can drastically change in response to physiological changes or specific cytokine stimulation, leading to alterations in expression levels and changes in the phosphorylation of the isoforms, resulting in distinct cell responses [2, 3].

In order to fully understand STAT3 function under physiological and pathological conditions, it is crucial to elucidate the specific roles of STAT3α and STAT3β together with the mechanisms that regulate their expression, activation and the interplay between them. Such an in-depth understanding will help in the design of specific drugs which may aim to alter the function of one of the STAT3 isoforms and not the other, possibly leading to more accurate diagnosis and efficient therapy.

In this study we examined the roles of STAT3 isoforms using specific siRNAs that target either STAT3α or STAT3β. We used the MDA-MB-231 cell line which represents an aggressive and mortal subtype of breast cancer, in which STAT3 is overexpressed and constitutively activated [14]. Our results show that each of the isoforms affects the activation (i.e., phosphorylation) of the other isoform and leads to changes in the outcome of the cells. We therefore conclude that both STAT3α and STAT3β play a crucial role in the function of STAT3 and must be addressed when planning STAT3-based therapies.

## RESULTS

### Silencing STAT3α or STAT3β affects STAT3 activation

**Figure 2:**
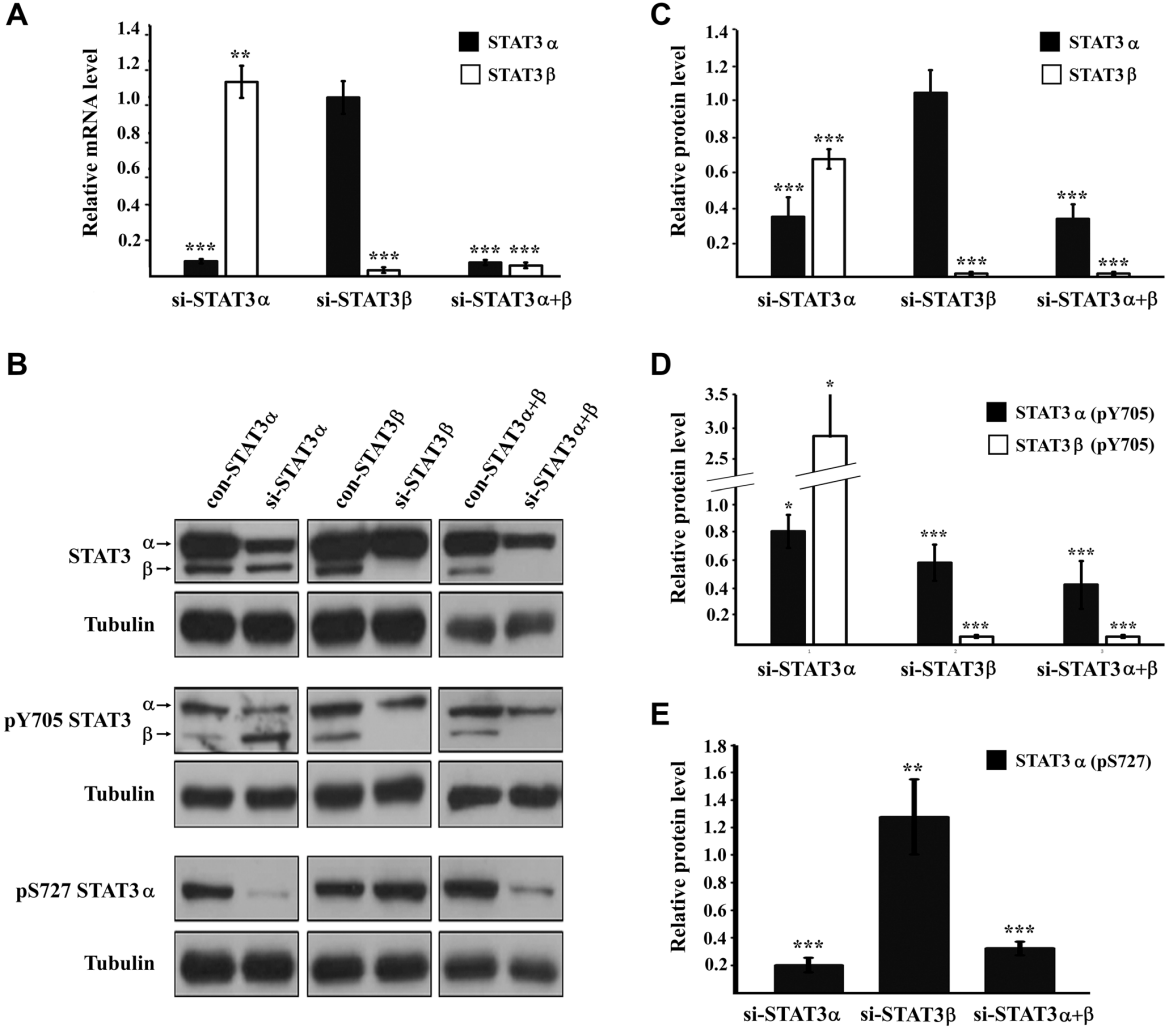
STAT3 expression and activation upon mRNA silencing (**A**) Relative expression of the mRNA of STAT3 isoforms evaluated by RQ-PCR. Results were normalized to those obtained upon transfection with the relevant negative control siRNA that was set to 1. (**B**) Western blot analysis of cells transfected with the indicated siRNA. Proteins were probed with anti-STAT3, anti-pY705 STAT3, anti-pS727 STAT3 or anti-αTubulin antibody. (**C**) Relative protein quantification of STAT3 in (B). (**D**) Relative protein quantification of pY705 STAT3 in (B). (**E**) Relative protein quantification of pS727 STAT3 in (B). All protein quantifications were performed with the ImageJ software. The protein amount obtained in the relevant control transfection was set to1. Results are the mean of at least three repeats of each experiment ± SD. ^*^ *P* ≤ 0.05; ^**^ *P* ≤ 0.01; ^***^ *P* ≤ 0.001.

To study the role of the alternatively-spliced STAT3 isoforms (Figure 1) in cancer cells we silenced the mRNA expression of either STAT3α (si-STAT3α), the main isoform, or STAT3β (si-STAT3β) or both (si-STAT3α+β) in MDA-MB-231 breast cancer cells using specific siRNA molecules. RNA analysis of the specific isoforms confirmed that the silencing was isoform-specific (Figure 2A). Western blot analysis revealed that transfecting the cells with si-STAT3α resulted in a 66% decrease in the amount of the STAT3α protein. However, we were also able to detect a 34% decrease in the amount of the STAT3β protein although STAT3β mRNA levels remained almost unchanged. Following silencing with si-STAT3β there was a sharp decrease in the amount of STAT3β (the protein could no longer be detected) while the amount of STAT3α was not affected. Transfection with si-STAT3α+β resulted in a ~70% decrease in STAT3α and a sharp decrease in the amount of STAT3β (Figure 2B, 2C). The activation of STAT3α and STAT3β proteins was studied by determining the level of tyrosine phosphorylation (pY705) on each of the isoforms. Upon silencing of STAT3α, there was only a 20% reduction in the amount of pSTAT3α (Y705) molecules. Surprisingly, silencing of STAT3α caused a 3-fold increase in the amount of pSTAT3β (Y705). The effect of si-STAT3β on pSTAT3β (Y705) could not be determined due to the very low protein levels upon silencing, however we detected a 40% decrease in the amount of pSTAT3α (Y705) although STAT3α protein levels remained unchanged. Si-STAT3α+β caused a 2-fold decrease in pSTAT3α (Y705) (the level of pSTAT3β (Y705) could not be determined) (Figure 2B, 2D). In addition to pY705, STAT3α can also be phosphorylated on the serine residue (pS727). This residue does not exist in the STAT3β protein. Western blot revealed that si-STAT3α caused an 80% decrease in pSTAT3α (S727) while si-STAT3β caused a 28% increase in the phosphorylation of this residue in STAT3α. Si-STAT3α+β caused a 70% decrease, in pSTAT3α (S727) levels (Figure 2B, 2E). Taken together these results imply that there is an interplay between these proteins at both the expression and activation levels. In order to evaluate the relative levels of STAT3α and STAT3β we calculated the ratio between the two proteins (STAT3α:STAT3β). With no treatment, the STAT3α: STAT3β ratio was 4:1. Silencing STAT3α reduced the ratio to 2:1 and upon silencing of STAT3β the ratio increased to at least 10:1. Silencing of both isoforms (si-STAT3α+β) resulted in a 5:1 ratio.

To further validate these results, STAT3 expression was studied in an additional breast cancer cell line. We previously published that silencing of STAT3 isoforms in MCF7 cells, originating from carcinoma breast cancer, resulted in specific silencing of each of the isoforms [15]. In the present study we expanded our analysis to study the activation of the STAT3 proteins in this cell line upon STAT3 silencing. Western blot analysis revealed that S727 phosphorylation in MCF7 cells results in a similar effect as in the MDA-MB-231 cells: silencing STAT3α resulted in a decrease in pS727 by 67% while silencing STAT3β resulted in a 31% increase (Supplementary Figure 1). Unfortunately, we could not study phosphorylation of Y705 since it occurs at very low levels in MCF7 cells and therefore very hard to detect [16]. Taken together, these results confirm that the phenomenon of one STAT3 isoform affecting the activation of the other STAT3 isoform is not specific to only one cell line, but is a more general one.

### STAT3α and STAT3β affect differently the gene expression of cytokines

**Figure 3:**
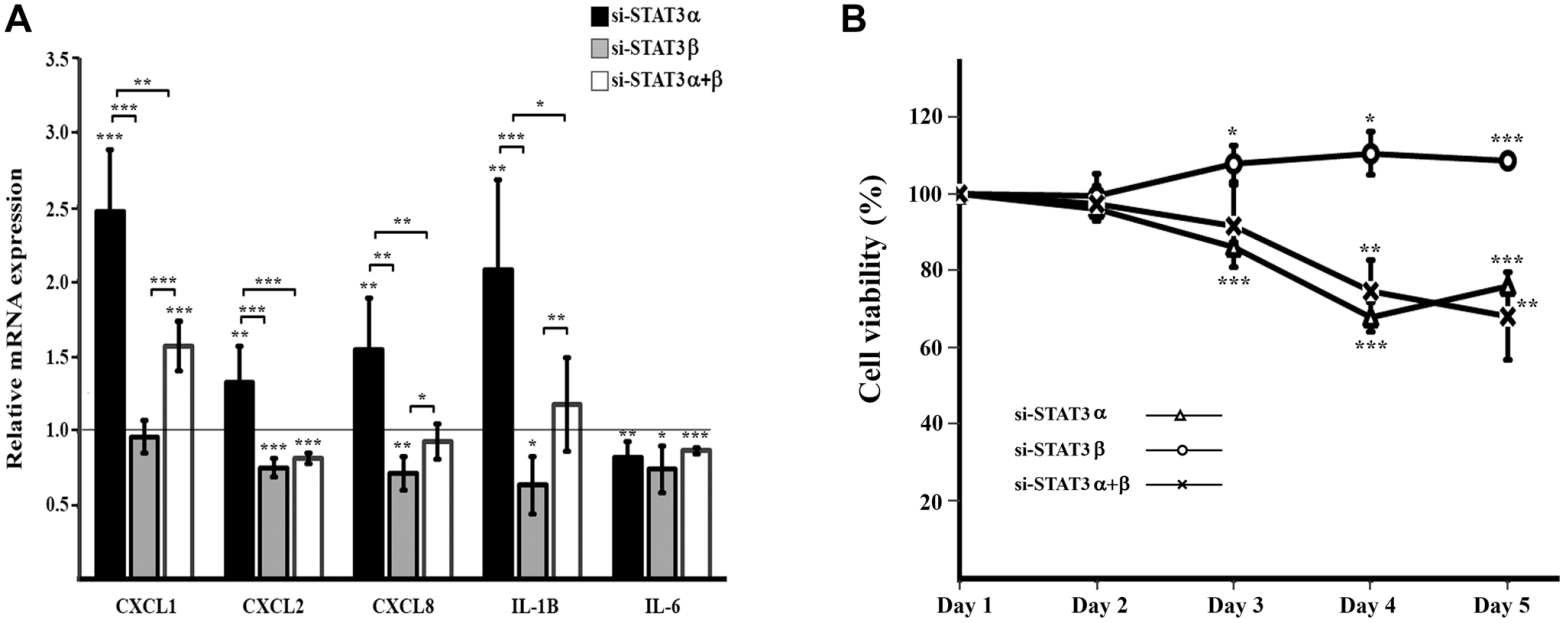
Cytokine expression and cell viability upon mRNA silencing of STAT3 isoforms (**A**) Relative quantification (RQ) of mRNA expression of cytokines upon mRNA silencing of STAT3 isoforms. Results were normalized to those obtained upon transfection with the relevant negative control siRNA that was set to 1. Results are the mean of at least three repeats of each experiment ± SD. ^*^ *P* ≤ 0.05; ^**^ *P* ≤ 0.01; ^***^ *P* ≤ 0.001. (**B**) Cell viability upon mRNA silencing of STAT3 isoforms evaluated by XTT assay, was performed over five days following transfection. Each time point was normalized to the measurement on day 1 and cellular growth was compared between treated cells to their respective control cells. Results are the mean of at least three repeats of each experiment ± SD. ^**^ *P* ≤ 0.01; Results are the mean of at least three repeats of each experiment ± SD. ^*^ *P* ≤ 0.05; ^**^ *P* ≤ 0.01; ^***^ *P* ≤ 0.001.

STAT3 plays a key role in the inflammatory response. We examined the mRNA expression of several cytokines upon silencing of the different STAT3 isoforms. We observed that silencing STAT3α caused an increase in the mRNA levels of CXCL1, CXCL2, CXCL8 and IL1B. However, an opposite outcome was observed upon silencing of STAT3β which caused a decrease in CXCL2, CXCL8 and IL1B expressions (no effect was seen for CXCL1). Furthermore, silencing both isoforms (si-STAT3α+β) did not affect the expressions of CXCL8 and IL1B but did cause an increase in CXCL1 and a decrease in CXCL2 mRNA levels. IL-6 levels decreased upon all treatments (si-STAT3α, si-STAT3β and si-STAT3-α+β) (Figure 3A).

### Cell viability is decreased by silencing STAT3α or STAT3α+β, but is increased by silencing STAT3β

Changes in STAT3 activation and in the gene expression of cytokines, which we observed upon silencing of STAT3 isoforms, can affect cell proliferation. In order to examine the distinctive roles of STAT3α and STAT3β on cell proliferation we preformed XTT assays on the treated cells and their controls. Metabolic activity of the cells was followed during five days after transfection (Figure 3B). The si-STAT3α and si-STAT3α+β treatments caused a reduction in cell viability whereas si-STAT3β caused an increase in cell viability. These results emphasize, once again, the different roles carried out by the STAT3α and STAT3β isoforms.

### Different outcomes in cell migration upon silencing of STAT3 isoforms

**Figure 4:**
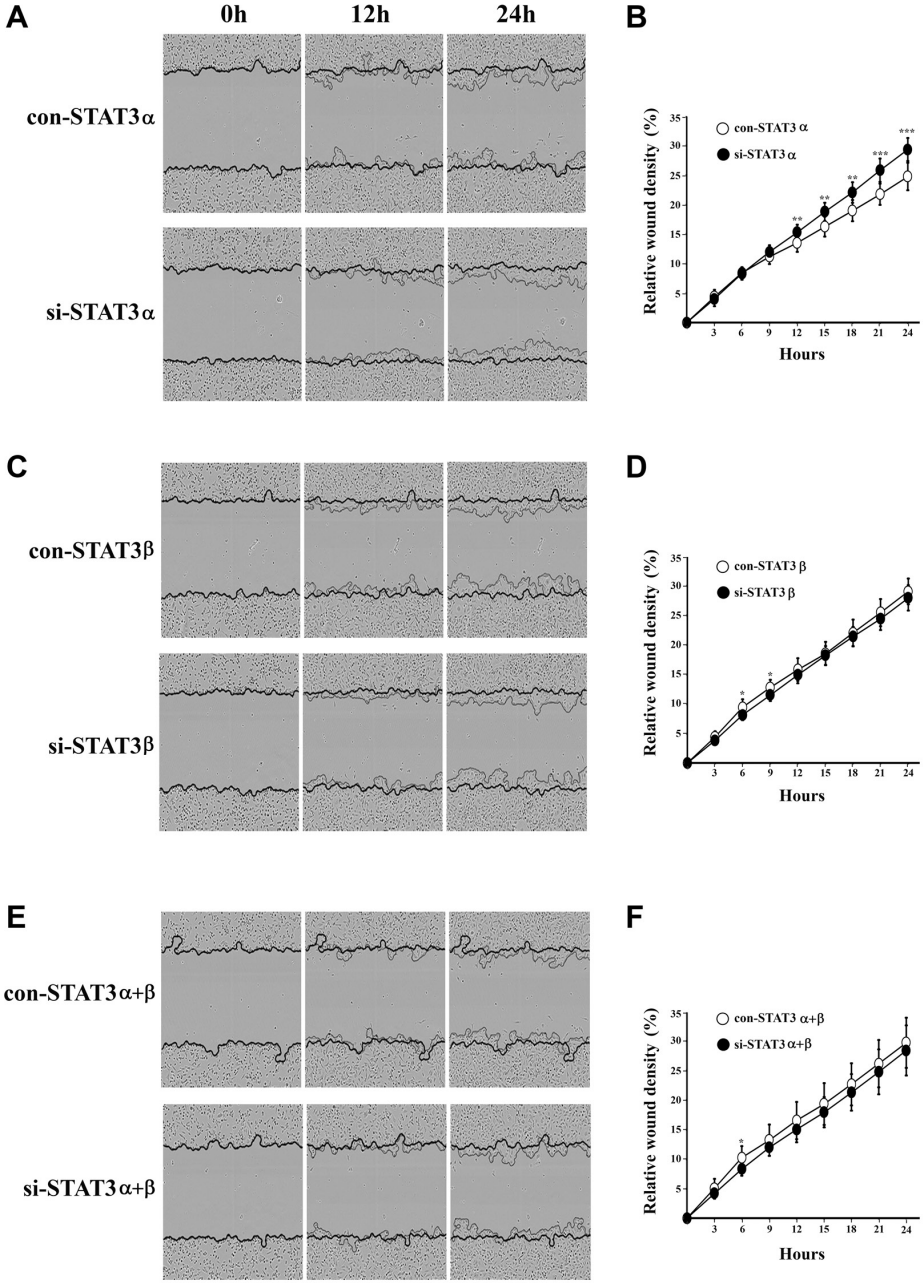
Cell migration upon mRNA silencing of STAT3 isoforms was measured using the Incucyte scratch wound healing assay. Images were taken every 45 minutes during 24 hours. Relative Wound Density (RWD) was calculated using the Incucyte Scratch Wound Analysis Software, to quantify migration. On the left panel are representative images of cell migration for each treatment ((**A**) si-α (**C**) si-β (**E**) si-α+β) which were taken at time 0 hours (when the scratch was performed), 12 and 24 hours. In the right panel are graphs that represent the RWD for each treatment ((**B**) si-α (**D**) si-β (**F**) si-α+β) measured for 24 hours (24–48 hours from transfection). All measurements were performed in triplicates and each experiment was repeated three times ± SD. ^*^ *P* ≤ 0.05; ^**^ *P* ≤ 0.01; ^***^ *P* ≤ 0.001.

The MDA-MB-231 cell line represents an aggressive and mortal subtype of breast cancer. Recent studies have shown that STAT3 plays a role in the tumorigenesis of those cells [14]. One of the major hallmarks of tumorigenesis is the ability of the cells to migrate. In order to study the contribution of the STAT3α and STAT3β isoforms to cell migration, we followed the closing of a scratch wound during 24 hours in MDA-MB-231 cells transfected with either si-STAT3α or si-STAT3β or both. Silencing of both isoforms (si-STAT3α+β) slightly decreased cell migration when compared to the control-transfected cells. However silencing of STAT3α resulted in a significant increase whereas silencing of STAT3β had almost no effect on cell migration (Figure 4A–4F).

## DISCUSSION

The opposing roles of oncogenic STAT3α and tumor suppressor STAT3β have led researchers to suggest that altering the ratio between them may lead to improved outcomes for the cells and patients. Our study reveals that silencing STAT3α expression leads to increased activated STAT3β levels, providing a mechanism for favorably shifting the isoform ratio without exogenous DNA insertion. Previous studies involving STAT3 isoforms have relied on exogenic copies of STAT3α or STAT3β, which may yield artifacts in the results. To more accurately mimic physiological changes, we manipulated endogenous STAT3 isoform expression and measured outcomes. Our findings strongly support the need to consider both isoforms in evaluating and treating patients based on STAT3 expression and activation. Inhibition of one STAT3 isoform, while sparing the other, may improve cancer treatment outcomes.

Our findings indicate that silencing STAT3α mRNA not only reduces the amount of STAT3α protein but also leads to a decline in STAT3β protein, despite unaffected STAT3β mRNA levels. However, the decrease in STAT3β is less pronounced compared to STAT3α, causing a change in the protein ratio from 4:1 to 2:1. These results suggest that STAT3α may have a role in maintaining STAT3β protein levels.

**Figure 5:**
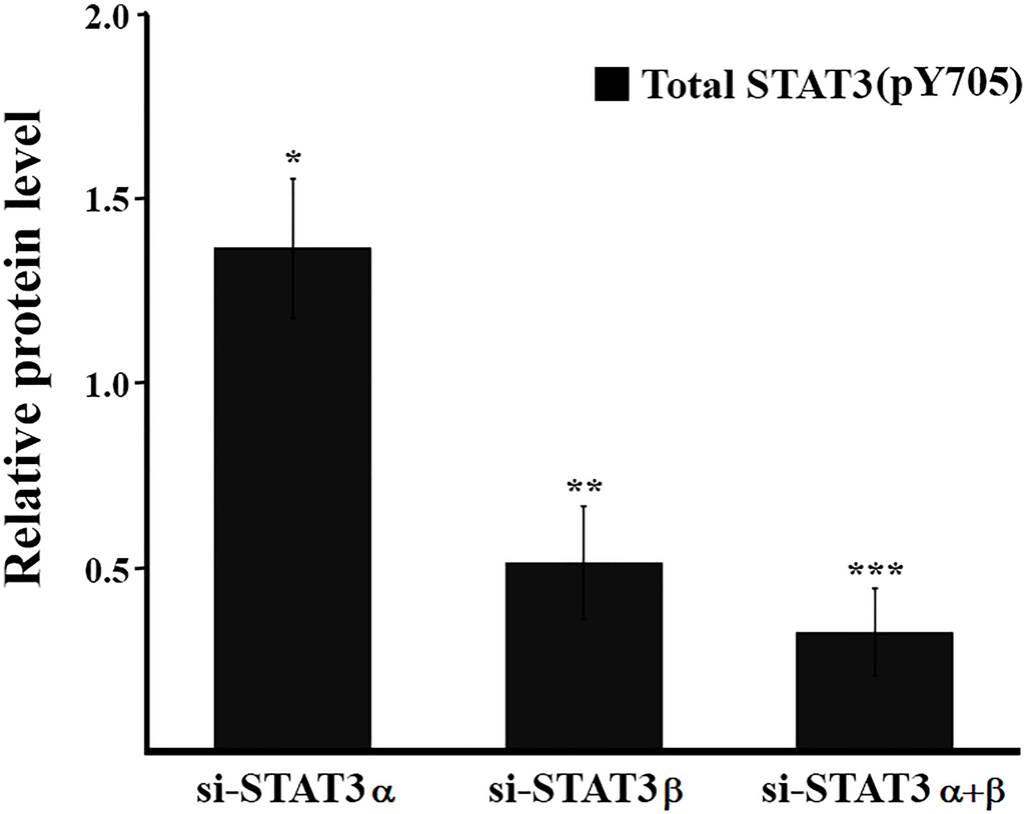
Estimation of the relative amount of total pSTAT3 (Y705). Estimation of the relative protein quantification was performed for total pSTAT3 (Y705) (pSTAT3α (Y705) + pSTAT3β (Y705)) with the ImageJ software from results shown in Figure 2B. The total protein amount in the relevant control was set to 1. Calculations were performed on the results of at least three technical repeats of each biological experiment ± SD. ^*^ *P* ≤ 0.05; ^**^ *P* ≤ 0.01; ^***^ *P* ≤ 0.001.

Phosphorylation of STAT3 on tyrosine 705 (Y705) is considered a marker for STAT3 activation and is well documented across multiple cancer types. It often correlates with a poor prognosis and accelerated disease progression [3]. Therefore, inhibiting pY705 is considered an attractive strategy for anti-cancer therapies. However, we show, that under certain conditions, an increase in total pSTAT3 (pSTAT3α and pSTAT3β) is not necessarily coupled with a bad outcome: in our system, while silencing STAT3α mRNA caused a decrease in the amount of both STAT3α and STAT3β proteins, it also led to a 37% increase in total pY705 STAT3 (Figure 5) and reduced cell viability. The increase in the total levels of pY705 STAT3 is attributed mainly to the 3-fold higher levels of pSTAT3β (Y705) and suggests that STAT3α may play a role in inhibiting the activation of the tumor suppressor, STAT3β. Additionally, silencing STAT3α mRNA resulted in a 65% decrease in the amount of the STAT3α protein but only a 20% decrease in pSTAT3α (Y705) levels. Similar observations were made in other breast cancer cell lines [16] and in esophageal squamous cell carcinoma (ESCC) where STAT3β overexpression protected pSTAT3α (Y705) from dephosphorylation [12]. As a result of STAT3α mRNA silencing the ratio of pSTAT3α to pSTAT3β shifted from 3:1 to 2:3, increasing the likelihood of forming pSTAT3α (Y705): pSTAT3β (Y705) heterodimers and/or pSTAT3β (Y705) homodimers, which have been shown to be more stable and can lead to improved cell fate and better prognosis [17, 18].

STAT3 can be activated through phosphorylation on the S727 residue, which is exclusive to the STAT3α isoform. Studies have shown that pSTAT3α (S727) preferentially localizes in the mitochondria, where it regulates the electron transport chain [19]. Kinases ERK1 and ERK2 contribute to this phosphorylation [6, 20]. Interestingly, recent research has revealed that high levels of STAT3β can hinder ERK1/2’s ability to phosphorylate STAT3α at S727 [21]. Therefore, removing STAT3β may render STAT3α more susceptible to ERK1/2, resulting in increased levels of pSTAT3α (S727). Indeed, our study demonstrated an increase in pSTAT3α (S727) levels upon silencing of STAT3β, which could potentially impact cell function due to the accumulation of active STAT3α in the mitochondria [1]. Further studies are required to determine the specific effects of phosphorylation at the S727 residue upon STAT3β silencing.

To investigate how changes in STAT3α and STAT3β levels and activation affect gene transcription patterns, we examined the mRNA levels of several genes involved in immune system recruitment and inflammation. CXCL1, CXCL2, CXCL8 and IL1B exhibited elevated mRNA levels upon silencing of STAT3α and decreased levels upon silencing of STAT3β. The differential transcription pattern upon silencing of each isoform suggests that STAT3β also plays a role in the expression of these genes. We propose several possible mechanisms, 1. STAT3β being responsible for the transcription of these genes and/ or STAT3α responsible for their repression 2. pSTAT3β having greater DNA-binding activity than pSTAT3α, leading to an induction in the transcription of STAT3β-regulated genes [22, 23] 3. As mentioned above, the abundance of pSTAT3β (Y705) results in a greater probability to form stable heterodimers [2, 3, 13, 22, 24, 25]. The intermediate transcription pattern observed upon silencing of both isoforms suggests that both isoforms play a role in gene transcription. These findings have implications for both autocrine and paracrine activities, affecting cell signaling and shaping the tumor microenvironment. The outcomes of our study align with previous researches that have proposed an opposing function of STAT3β to STAT3α [1–3, 13, 26, 27]. However, we cannot compare our findings from silencing both isoforms using separate siRNAs with the outcomes of studies that used a mutual region-directed siRNA to silence STAT3. Our “double treatment” resulted in a highly effective mRNA silencing of each isoform whereas using a common siRNA that could randomly hybridize with either STAT3α or STAT3β transcripts, which are present in different ratios in the cell [28] would lead to a different STAT3α/STAT3β ratio than what we observed in our si-STAT3α+β treatment. As opposed to the differential effects that we observed in the transcript levels of CXCL1, CXCL2, CXCL8 and IL1B upon si-STAT3α or si-STAT3β, we found IL6 transcription to be reduced upon all our treatments. Several studies reported that STAT3 is part of a positive feedback loop which plays a role in promoting interactions between non-immune and immune cells [29]. Our results show that both STAT3 isoforms play a role in IL6 transcription.

STAT3 plays an important role in promoting cell survival [30, 31]. Our study found that silencing STAT3α reduces cell viability, while silencing STAT3β increases it, suggesting opposing roles for the two isoforms. While STAT3 is known to contribute to cell viability through its role as a transcription factor in regulating cell cycle genes [2, 6], our results suggest that activation of STAT3 via pY705 is not the only trigger for proliferation (e.g. decreased cell viability when pSTAT3 (Y705) levels are high (silencing of STAT3α) or increased cell viability when pSTAT3 (Y705) levels are low (silencing of. STAT3β). CXCL1, CXCL2, CXCL8, and IL1B belong to the TNF signaling pathway and their elevated expression has been shown to be involved in the induction of necroptosis [13, 32]. This indeed can explain the lower cell viability obtained upon silencing STAT3α or STAT3α+β. Silencing STAT3β, on the other hand, reduced necroptosis and increased cell viability. It’s worth noting that many studies on pSTAT3 (Y705) do not differentiate between the two isoforms, which could lead to misconceptions. We could not determine the exact mechanism underlying changes in cell viability, leaving open the possibility of multiple mechanisms. Recent studies have identified various STAT3 functions that influence cell viability independently of nuclear gene transcription, including those dependent on phosphorylation at serine S727, which has been linked to cell proliferation [1, 6, 33]. Our results indicate that phosphorylation at S727 may play a role in regulating cell viability, as silencing STAT3α and α+β led to reduced pSTAT3α (S727), while silencing STAT3β resulted in increased pSTAT3α (S727). However, additional experiments are needed to fully elucidate the mechanisms responsible for cell viability changes upon altering STAT3 isoform levels. Our findings are consistent with previous research demonstrating that high levels of pSTAT3β (Y705) inhibit cell proliferation and induce cell death [13], and that overexpression of STAT3β in cancer cells results in reduced proliferation, increased apoptosis, and tumor regression [27, 34, 35].

Cancer cells have the ability to invade and form metastases, a process which is associated with epithelial-mesenchymal transition (EMT). Constitutively dimerized forms of STAT3 play a key role in promoting invasion and metastasis [36, 37]. Indeed, we show that when pSTAT3 (Y705) levels are high in the cells (e.g. upon STAT3α silencing) cell motility is increased while silencing STAT3β or STAT3α+β (resulting in decreased levels of pSTAT3 (Y705)) has no effect. Only the treatment of si-STAT3α resulted in an increase in the total amount of pSTAT3 (Y705) probably leading to an excess of stable heterodimers. Our attempts to look for differential changes in the mRNA expression of genes known to be involved in EMT such as Vimentin, ZEB1, FIN1, Snail1, Snail2 and MMP1, were unsuccessful (Supplementary Figure 2 and Supplementary Table 1). Further experiments are needed to elucidate the exact mechanism involved in changes in cell motility.

Our study emphasizes the importance of distinguishing between STAT3α and STAT3β proteins and their active forms when discussing STAT3-related cancer diagnosis and therapy. Referring to STAT3 as a single protein can lead to wrong conclusions, as they have different functions. Current STAT3 inhibitors target both isoforms, but this approach should be revised for better patient care. We present an endogenous mechanism that can shift the balance in a favorable direction, and we suggest developing treatments that mimic this mechanism could lead to new avenues for cancer therapy.

## MATERIALS AND METHODS

### Cell culture

MDA-MB-231 (RRID: CVCL_0062) cells, from a human breast cancer cell line, isolated from a female with adenocarcinoma, were obtained from the American Type Culture Collection (ATCC). The cell line was confirmed to be free of Mycoplasma on a regular basis using PCR Mycoplasma kit (Biological Industries, 20-700-20). Cells were cultured in Dulbecco’s modified Eagle’s medium (DMEM) (Biological Industries, 01-055-1A) supplemented with 10% fetal bovine serum (FBS) (Biological Industries, 04-007-1A), 1% penicillin: streptomycin (Biological Industries, 03-031-5C) and 1% glutamine (Biological Industries, 03-020-1B), at 37°C in a humidified incubator with 5% CO_2_.

### siRNA of STAT3α and STAT3β

siRNAs are specific for each of the STAT3 isoforms (Ambion, Pleasanton, CA, USA) (5′ to 3′):

si-STAT3α-sense: GCAAUACCAUUGACCUGCCtt;
si-STAT3α-antisense: GGCAGGUCAAUGGUAUUGCtg;
si-STAT3β-sense: GUGUGACACCAUUCAUUGAtt;
si-STAT3β-antisense: UCAAUGAAUGGUGUCACACag;
siRNA negative control (si-CON) (Ambion, Cat. #AM4635).

### Transfection of siRNA

MDA-MB-231 cells were transfected using Lipofectamin 3000^®^ (Invitrogen, Carlsbad, CA, USA, L3000-008). Briefly, 2.5 × 10^5^ cells were seeded in six-well plates. After 24 hours siRNA was added to the following final concentrations: si-STAT3α 0.02 μM; si-STAT3β 0.01 μM; si-CON was added to a similar concentration as the relevant si-STAT3. Transfection was continued according to the manufacturer’s protocol. The expressions of mRNA and proteins were analyzed 48 hours following transfection. All experiments were repeated at least three times.

### RNA extraction, cDNA preparation and PCR analysis

Total RNA was extracted using TRI Reagent^®^ (Sigma/Merck, Darmstadt, Germany, T9424) according to the manufacturer’s protocol. cDNA was prepared from 2 μg RNA with the High-Capacity cDNA Reverse-Transcription Kit (Applied Biosystems, Vilnius, Lithuania).

Relative quantitative (RQ)-PCR was performed with the TaqMan^®^ (AB-4444557) or SYBR Green^®^ (Ref: 4385612) Fast Advanced Master Mixes (Applied Biosystems) with 0.5 μM of each primer. Probes (5’FAM, 3’BHQ) were used at the following final concentrations: STAT3α 0.25 μM; STAT3β 0.25 μM and ABL 0.125 μM.

The primers used in our study (5′ to 3′):

STAT3α-F: TGACACCAACGACCTGCAG
STAT3α-R: CAGCACCTTCACCATTATTTCCA
STAT3α-probe: CCCCGCACTTTAGATTCATTGATGCAGT
STAT3β-F: GCCCCATACCTGAAGACCAA
STAT3β-R: TCAGCACCTTCACCATTATTTCC
STAT3β-probe: TTTATCTGTGTGACACCATTCATTGATGCAGTT
ABL-F: TGGAGATAACACTCTAAGCATAACTAAAGGT
ABL-R: GATGTAGTTGCTTGGGACCCA
ABL-probe: CCATTTTTGGTTTGGGCTTCACACCATT
CXCL1-F: GCAGCAGTCAGTGAGTCTCTTC
CXCL1-R: GGGGACTTCACGTTCACACT
CXCL2-F: CAAACCGAAGTCATAGCCAC
CXCL2-R: GGAACAGCCACCAATAAGCT
CXCL8-F: GTCTGGACCCCAAGGAAAAC
CXCL8-R: TTCTTGGATACCACAGAGAATGAA
IL-1B-F: TCCAGGGACAGGATATGGAG
IL-1B-R: TCTTTCAACACGCAGGACAG
IL-6-F: CGGGAACGAAAGAGAAGCTC.
IL-6-R: AGGCAACACCAGGAGCAG.

All primers were designed to amplify mature mRNA only. The ABL gene was used as a reference gene. All reactions (10 μL) were performed in triplicates on the Applied Biosystems StepOne™ machine using the StepOne v2.3 software. RQ analyses were performed with the ΔΔCT method. Each experiment was performed at least three times. Results were normalized to those obtained upon transfection with the relevant si-CON which was set to one.

### Cell lysis and western blot analysis

Proteins were extracted using RIPA buffer (Sigma/Merck, Darmstadt, Germany, R0278) supplemented with a protease inhibitor (Roche, Basel, Switzerland, 11836170001) and phosphatase inhibitors (Sigma/Merck, Darmstadt, Germany, P5726, P0044). Following separation on a 7.5% or 10% SDS-PAGE, proteins were transferred to a nitrocellulose membrane followed by staining with a primary antibody overnight at 4°C, washed and incubated with the appropriate secondary antibody for 45 minutes at room temperature. Specific reactive bands were detected with horseradish peroxidase-conjugated secondary antibodies by enhanced chemiluminescence (Cyanagen, 9470XLS0700250). Quantification of proteins was performed with the ImageJ software (National Institute of Health, USA). The antibodies used were as follows: anti-STAT3 1:1000 (Cell Signaling Technology (CST), USA, 124H6) which detects both STAT3 isoforms, anti-pSTAT3 Y705 1:1000 (CST, #9145) which detects both STAT3 isoforms, anti-pSTAT3 S727 (CST, #9134), anti-Tubulin 1:30000 (Abcam).

### Cell proliferation assay (XTT)

Cell viability was measured with a cell proliferation kit (XTT, 20-300-1000, Biological Industries). 2.5 × 10^5^ cells were seeded in six-well plates. For the XTT assay, six hours following transfection, live cells (approximately 8 × 10^3^ cells) were re-seeded in 96-well plates with DMEM medium in a final volume of 100 μl. Plates were incubated at 37°C in a 5% CO_2_ incubator. XTT assays were performed according to the manufacturer’s protocol. Absorbance was measured at 475 nm against a reference wavelength at 660 nm using a microplate reader (Infinite M200 PRO, Tecan). Cell viability was measured at the following time points: 24 h (day1), 48 h (day2), 72 h (day3), 96 h (day4) and 120 h (day 5) following transfection. Results were expressed as the percentage of XTT, (the absorbance of control cells was set as 100%). All measurements were performed in triplicates and each experiment was repeated at least three times. Each time point was normalized to the measurement on day 1 and cellular growth was compared between treated cell (si-STAT3α, si-STAT3β or si-STAT3α+β) and control cells (si-CON-α, si-CON-β or si-CONα+β).

### Scratch wound assay

2.5 × 10^5^ cells were seeded in six-well plates. Six hours following transfection, live cells were re-seeded in 96-well IncuCyte^®^ ImageLock Plates (Essen BioScience) at a confluency of approximately 75% with DMEM medium in a final volume of 100 μl. Plates were incubated overnight at 37°C in a 5% CO_2_ incubator. 24 hours following transfection, the wound maker tool (“IncuCyte^®^ wound maker”) was used to make scratch wounds and the media was replaced with 100 μl of fresh medium. Plates were incubated in the IncuCyte machine (Essen BioScience, USA) and images were taken every 45 minutes during 24 hours. Relative Wound Density was calculated using IncuCyte^®^ Scratch Wound Analysis Software to quantify migration. All measurements were performed in triplicates and each experiment was repeated three times.

### Statistical analysis

*T*-test was used to calculate statistical differences between two samples. Results are given as mean value + SD. A *P*-value of ≤ 0.05 was considered statistically significant.

## SUPPLEMENTARY MATERIALS



## Abbreviations

STAT3: Signal transducer and activator of transcription 3
ER: endoplasmic reticulum
TAD: transactivation domain
SH2: Src homology 2
RQ: Relative quantitative
