# Supplementary material for: Differential silencing of STAT3 isoforms leads to changes in STAT3 activation
Source: Oncotarget. 2023 Apr 24;14:366–76. doi: 10.18632/oncotarget.28412 (PMC12219269; doi:10.18632/oncotarget.28412)
Supplement: Supplementary file 1 [file oncotarget-14-28412-s001.pdf]

Differential silencing of STAT3 isoforms leads to changes in STAT3 activation

SUPPLEMENTARY MATERIALS

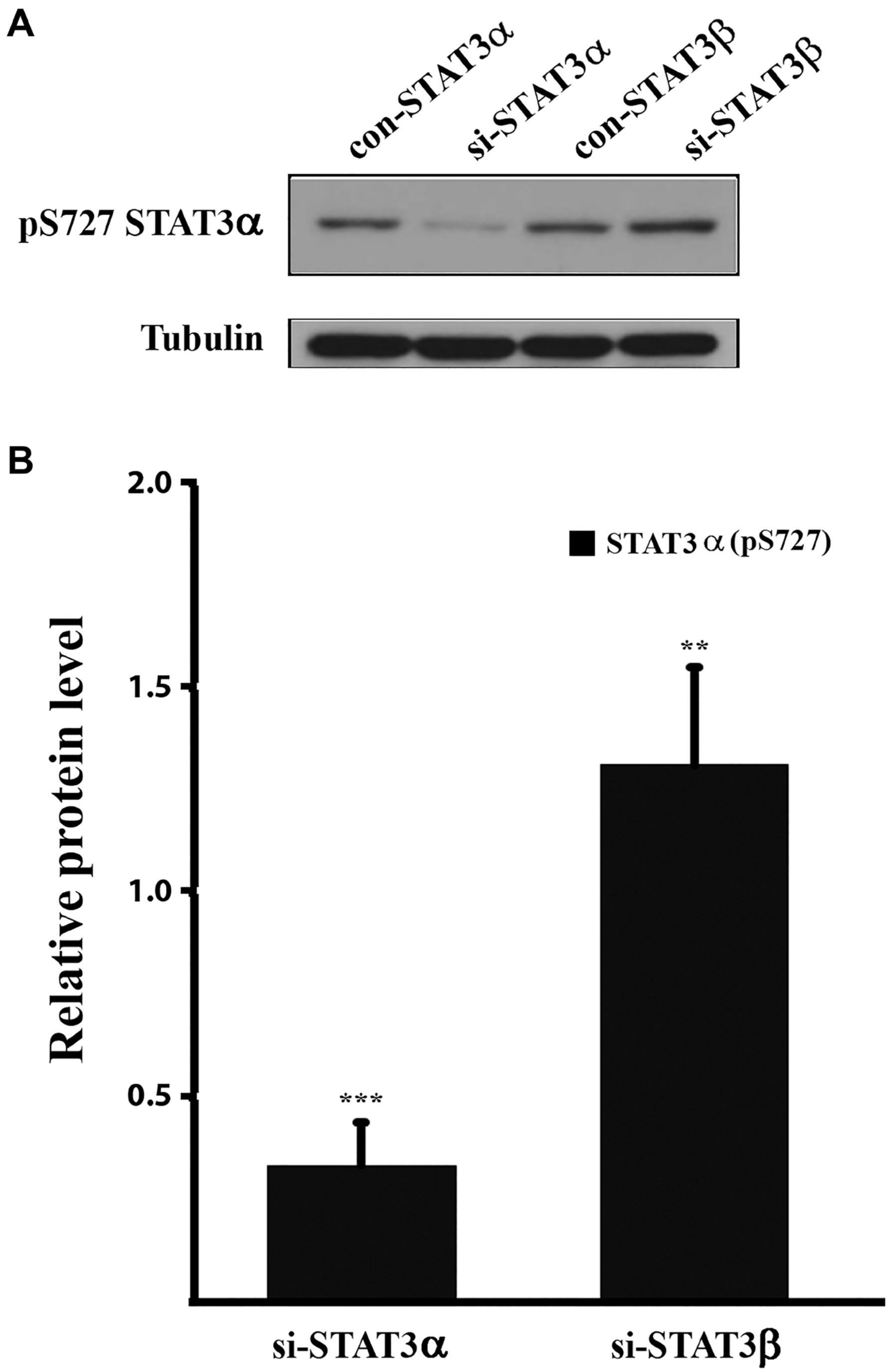

Supplementary Figure 1: STAT3 phosphorylation on residue S727 in MCF7 cells. (**A**) Western blot analysis of cells transfected with the indicated siRNA. Proteins were probed with anti-pS727 STAT3 or anti-αTubulin antibody. (**B**) Relative protein quantification of pS727 STAT3 in (A). Calculations were performed on the results of at least three technical repeats of each biological experiment ± SD. $^{**}P \leq 0.01$; $^{***}P \leq 0.001$.

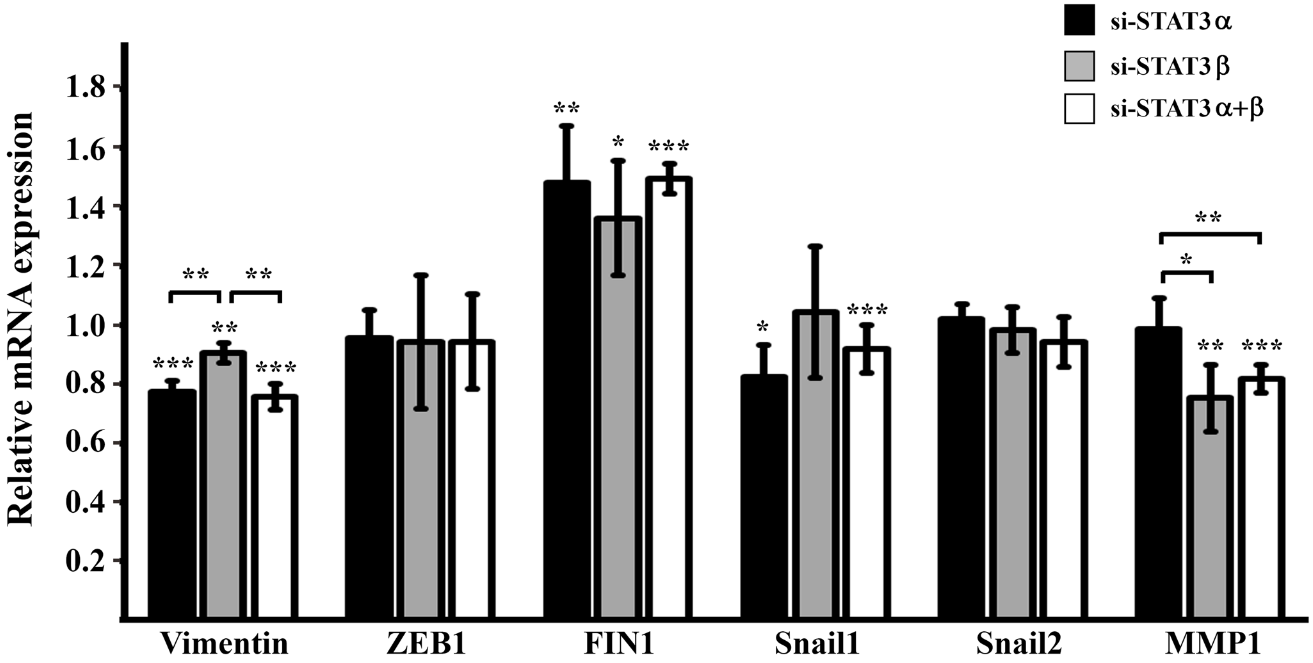

Supplementary Figure 2: Relative quantification (RQ) of mRNA expression of genes involved in EMT. The primers used for the RQ appear in Supplementary Table 1. Results were normalized to those obtained upon transfection with the relevant negative control siRNA that was set to 1. Results are the mean of at least 3 biological repeats and at least three technical repeats of each biological experiment ± SD. $^{*}P \leq 0.05$; $^{**}P \leq 0.01$.

Supplementary Table 1: Table of primers for Suppementary Figure 1

Primer	Sequence 5′ to 3′
Vimentin-F	GGGAGAAATTGCAGGAGGAG
Vimentin-R	ATTCCACTTTGCGTTCAAGG
ZEB1-F	GATGATGAATGCGAGTCAGATGC
ZEB1-R	ACAGCAGTGTCTTGTTGTTGT
FIN1-F	CGGTGGCTGTCAGTCAAAG
FIN1-R	AAACCTCGGCTTCCTCCATAA
Snail 1-F	TCGGAAGCCTAACTACAGCGA
Snail 1-R	AGATGAGCATTGGCAGCGAG
Snail 2-F	CGAACTGGACACACATACAGTG
Snail 2-R	CTGAGGATCTCTGGTTGTGGT
MMP-1-F	ACGAATTTGCCGACAGAGAT
MMP-1-R	GCAGCATCGATATGCTTCAC
ABL-F	TGGAGATAACACTCTAAGCATAACTAAAGGT
ABL-R	GATGTAGTTGCTTGGGACCCA
